# Supplementary material for: Adaptive transgenerational plasticity through priming in response to neighbor density in Mimulus platycalyx
Source: PLoS One. 2026 May 15;21(5):e0348265. doi: 10.1371/journal.pone.0348265 (PMC13178917; doi:10.1371/journal.pone.0348265)
Supplement: S1 File — (DOCX) [file pone.0348265.s001.docx]

**Supplemental Figures:**

**
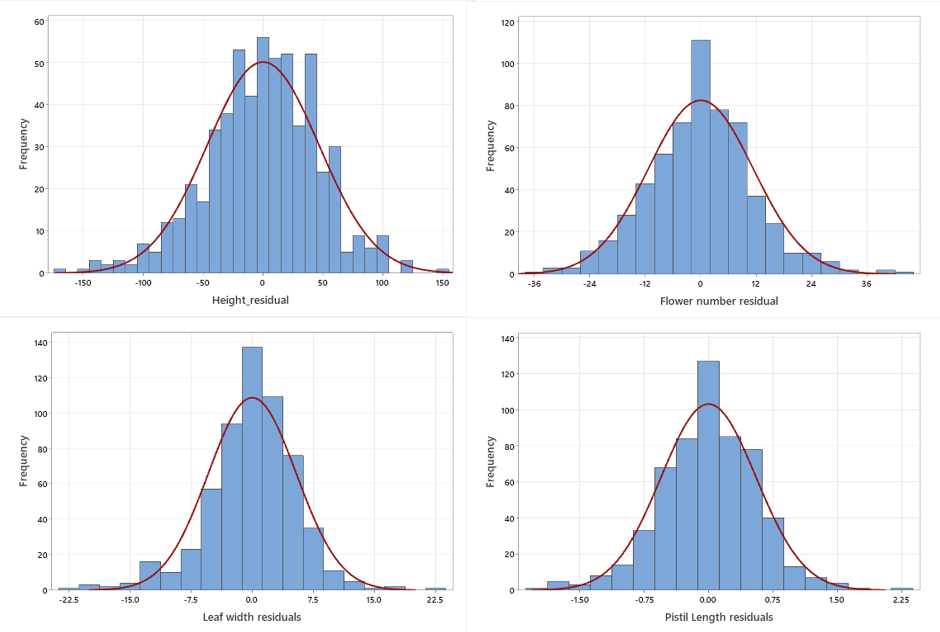
**

**Figure S0. The distributions of residuals from the generalized linear mixed model fits are reported for each trait in Table 1. The residual distributions for the model including covariates (Table 2) are not shown but they also exhibit close agreement to normality.**

**
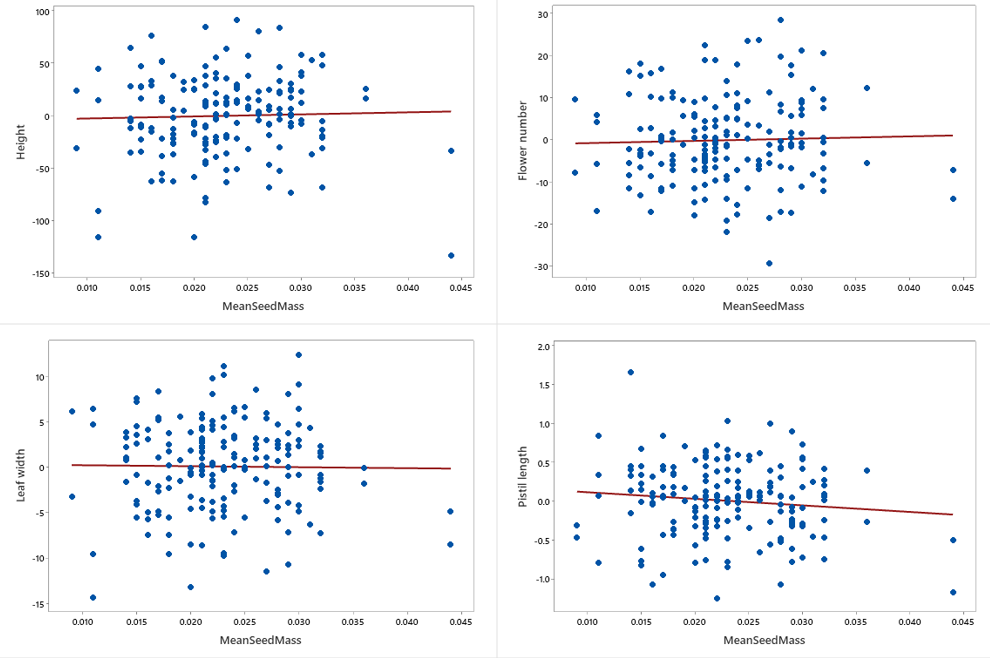
**

**Figure S1. The mean offspring trait value per family is reported as a function of mean parental seed mass for that family. Each point represents a family, and red lines show the linear regression fit. None of the slopes are significantly different from zero (p > 0.05), indicating no relationship between parental seed mass and offspring trait expression across families.**

**
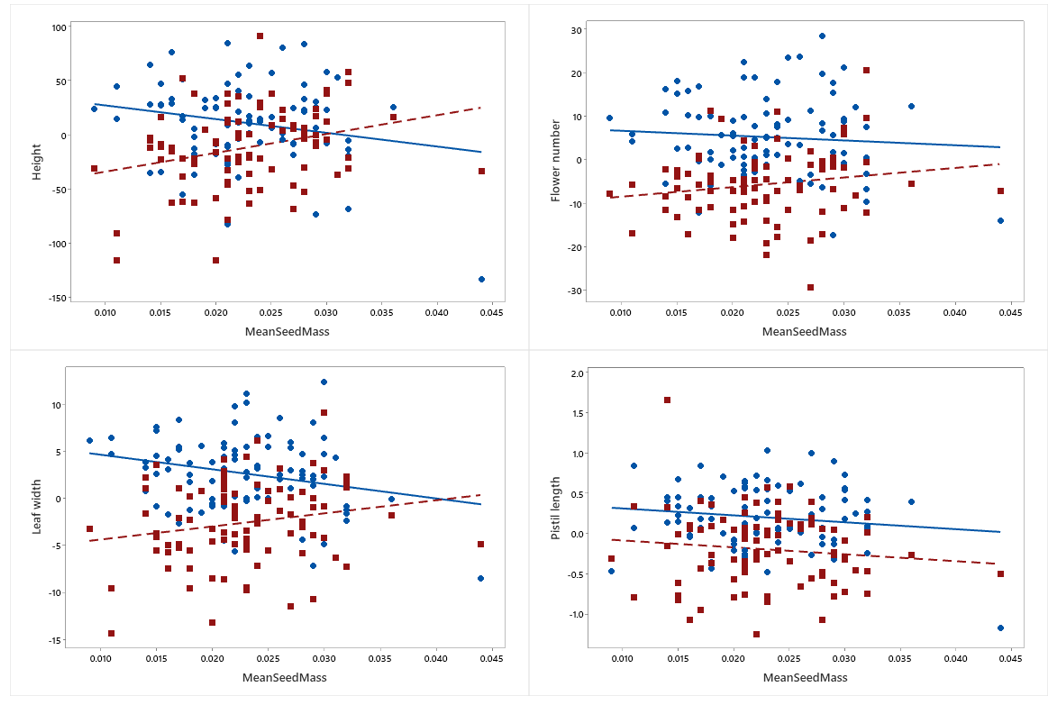
**

**Figure S2. Regressions of mean offspring trait values on mean parental seed mass are shown separately for Control (blue) and Crowded (red) offspring treatments. Each point represents a family, and lines indicate fitted linear regression models.**

**Supplemental table S1: Basic summary statistics for measured variables. Flower number is a count, all other variables in mm.**

| **Variable** | | **N** | **Mean** | | **Standard**  **Deviation** | |  |
| --- | --- | --- | --- | --- | --- | --- | --- |
| Flower Number | | 587 | 23.6 | | 20.03 | |  |
| Leaf Width | | 587 | 28.0 | | 8.39 | |  |
| Pistil Length | | 572 | 9.76 | | 0.78 | |  |
| Total Height | | 587 | 231 | | 76.10 | |  |
| Internode1 | 587 | | | 33.11 | | 17.19 | |
| Internode2 | 585 | | | 59.19 | | 18.17 | |
| Internode3 | 580 | | | 44.16 | | 10.75 | |
| Internode4 | 567 | | | 34.76 | | 8.77 | |
| Internode5 | 539 | | | 26.04 | | 8.91 | |
| Internode6 | 482 | | | 20.33 | | 8.10 | |
| Internode7 | 403 | | | 15.48 | | 6.86 | |
| Internode8 | 295 | | | 11.87 | | 6.80 | |
| Internode9 | 163 | | | 10.81 | | 5.60 | |
| Internode10 | 94 | | | 7.92 | | 4.82 | |
